# Malignant Transformation of a Vestibular Schwannoma Without Previous Radiation Exposure: Illustrative Case and Literature Review

**DOI:** 10.1055/a-2547-5320

**Published:** 2025-04-03

**Authors:** Chao Li, James Fowler, Kishore Balasubramanian, Kar-Ming Fung, Piao Zhe, William W. Wu

**Affiliations:** 1 Department of Neurosurgery, The University of Oklahoma Health Sciences Center, Oklahoma City, Oklahoma; 2 Department of Neurosurgery, Hartford Hospital, Hartford, Connecticut; 3 Texas A&M Health Science Center, Bryan, Texas; 4 Department of Pathology, Kaiser Fontana, Fontana, California

**Keywords:** vestibular schwannoma (VS), malignant peripheral nerve sheath tumor (MPNST), rhabdomyoblastic differentiation, malignant triton tumor, malignant transformation, cerebellopontine angle mass, immunohistochemistry, next-generation sequencing, S-100

## Abstract

**Background:**

Although malignant transformation of benign vestibular schwannoma (VS) preceded by irradiation has been well documented, few studies have demonstrated malignant transformation in the absence of radiation. Here, we present a rare case of the malignant transformation of a benign VS to a malignant peripheral nerve sheath tumor (MPNST) in the absence of prior irradiation. Additionally, we conducted a literature search to identify all other reported cases of MPNST arising from VS under similar conditions.

**Case Presentation:**

A 75-year-old female presented to the hospital with a 1-month history of left-sided facial numbness, loss of taste on the left side of her tongue, severe dysarthria, and recent-onset cranial nerve VI and VII palsies. MRI of the brain with and without contrast demonstrated an enlarging cerebellopontine angle mass and signs of brainstem compression. The patient underwent a left retrosigmoid craniotomy and surgical resection. Pathology and immunohistochemistry sequencing findings were consistent for MPNST with rhabdomyoblastic differentiation (malignant triton tumor). An outside review of the case by a large academic institution concurred with the diagnosis. The patient did not report any previous history of irradiation.

**Conclusion:**

A total of 11 cases, including ours, have appropriate S-100 immunochemical reactivity to confirm malignant transformation. Due to the limited number of reported cases of MPNST arising from VS without prior irradiation, information regarding pathogenesis and pathological diagnosis is scarce. We provide valuable additions to the literature, including next-generation sequencing data, to identify potentially targetable genetic changes and help elucidate the pathogenesis of MPNST.

## Introduction

Malignant peripheral nerve sheath tumor (MPNST), also known as neurogenic sarcoma, neurofibrosarcoma, or “malignant schwannoma,” is a malignant tumor of nerve sheath elements. Although MPNSTs most commonly originate from the nerves of the trunk or extremities, they may also arise from cranial nerves or the brain parenchyma. 1 2 3 4 5 6 7 8 9 The general incidence of MPNSTs has been reported to be 1 per million people per year; however, less than 5% of MPNSTs are intracerebral. 10 11 12 13 14 These malignancies can arise as a result of malignant transformation from pre-existing neurofibromas or, less commonly, from benign schwannomas. 13 Since the first reported case of an intracerebral MPNST, there has been increasing interest in this rare type of intracerebral tumor and its analogs. However, the histological diagnosis remains challenging. 13

Risk factors for MPNSTs include neurofibromatosis type 1 (NF1) and prior radiation. 12 14 15 Given the increasing number of patients receiving stereotactic radiosurgery for benign intracranial lesions, much of the literature over the past two decades has focused on radiation-induced malignancies. 16 The established risk of malignant transformation of a benign vestibular schwannoma (VS) after radiation is between 1 in 500 and 1 in 2,000. 16 17 18 While the malignant transformation of VSs to MPNSTs in the context of radiation history has been well documented, de novo transformation, in the absence of irradiation, is rare.

Given the paucity of literature on this subject, we present a rare case of an MPNST with rhabdomyolysis differentiation (malignant triton tumor) arising from benign VS without prior irradiation or history of neurofibromatosis. Further, we review the published literature to elucidate the characteristics of this malignancy and focus on the challenging histopathologic diagnosis. Our review focuses on cases published within the last two decades to capture the detailed immunochemical staining, such as S-100 protein reactivity, which is imperative in diagnosing MPNSTs secondary to malignant transformation.

## Illustrative Case

**Fig. 1:**
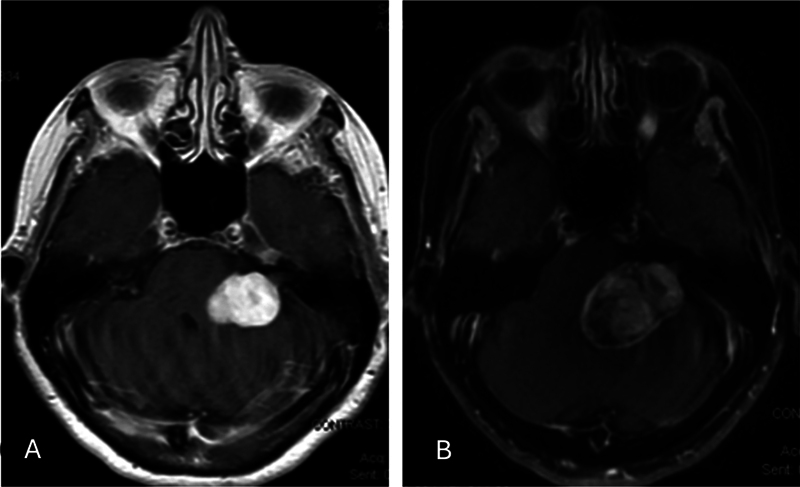
Initial contrast MRI of brain showing a homogeneously enhancing mass of the left cerebellopontine angle (CPA) in axial view (A). Follow-up contrast MRI of brain 3 weeks after the initial MRI demonstrating radiographic progression from a homogeneous enhanced lesion to heterogeneous enhanced lesion (B).

A 75-year-old female presented to the hospital with a 1-month history of left-sided facial numbness, loss of taste on the left side of her tongue, severe dysarthria, and gait instability. The patient reported a multi-year history of a known left-sided cerebellopontine angle (CPA) mass. However, previous radiographic studies were not available for review. Brain MRI, with and without contrast, at our institution showed a left CPA tumor with imaging characteristics consistent with VS ( Fig. 1A ).

After 3 weeks, she started to experience cranial nerve VI and VII palsies with the inability to close her left eyelid. Repeat MRI of the brain with and without contrast demonstrated a significant increase in size and enhancement and increased brainstem compression ( Fig. 1B ).

**Fig. 2:**
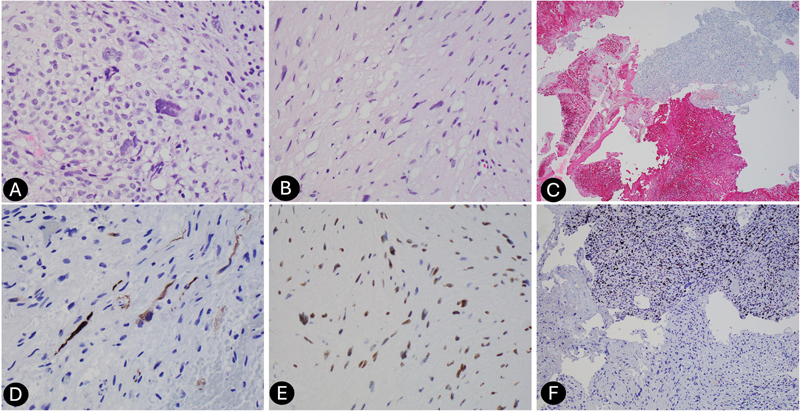
High power H&E stain with both benign and malignant parts of tumor (A and B). Low power S-100 benign part of tumor with diffuse stain and malignant part of tumor with S-100 scattered stain (C). Desmin immunohistochemical and H&E stain showing a rhabdomyoblastic component (D). High power image showing loss of H3K27me3 expression in malignant portions of the tumor compared with benign portions (E). High power Ki67 malignant part of tumor with increased proliferative index compared with benign part of tumor (F).

The patient subsequently underwent a left retrosigmoid craniotomy and surgical resection of the mass. Intraoperative findings showed no apparent border between the tumor capsule and cerebellum, with high vascularity in the anterior portion of the mass. A fragmented tan-pink irregular tissue admixed with red-clotted blood was seen on gross pathology. H&E-stained histologic sections revealed a hypercellular high-grade neoplasm with spindle and epithelioid components. Mitotic activity was elevated (21 per 10 high-power fields). Lower-grade areas were also present, supporting the development of a benign schwannoma ( Fig. 2A, B ). S-100 immunochemistry demonstrated scattered positivity in the high-grade areas and diffuse positivity in lower-grade regions ( Fig. 2C ). A rhabdomyoblastic component was identified by desmin immunohistochemical stain ( Fig. 2D ). Myogenin stain demonstrated weak expression in the rhabdomyoblastic component. H3K27me3 appeared lost in the high-grade tumor and retained in the lower-grade areas ( Fig. 2E ). INI1 expression was retained. Additional immunochemistry was negative for pancytokeratin and GFAP. The pathologic findings were consistent with MPNST with rhabdomyoblastic differentiation (malignant triton tumor). An outside review of the case by a large academic institution concurred with the diagnosis.

A tumor sample was submitted for next-generation sequencing (NGS) testing which analyzed 429 genes. NGS revealed EGFR amplification, EGFR vIII exon 2–7 deletion, NF2 exon 12 splice donor mutation, and TP53 mutations.

## Methods

A literature search was completed to identify all publications reporting intracerebral MPNSTs arising from VSs without prior irradiation. The National Library of Medicine (PubMed) and Google Scholar were searched. Search terms included “intracerebral neurogenic sarcoma,” “neurofibrosarcoma,” “schwannoma,” “MPNST,” and “malignant schwannoma.” All references were individually screened based on the titles and abstracts, and irrelevant studies were excluded. Links to “related studies” from PubMed and the bibliography from each included study were explored to ensure all relevant studies were captured. Individual entries were cross-checked to eliminate duplicate publications reported by more than one journal.

Our selection criteria for cases of intracerebral MPNSTs arising from VS included either (a) histologically confirmed cases of VS with a recurrent mass at the same site demonstrating MPNST or (b) the finding of a histologically malignant component within a VS on the same pathologic section. None of the included cases contained primary tumors elsewhere that may have metastasized to the VS. Cases with a prior history of radiation were excluded, as were cases with a prior history of VS that received radiation therapy.

Data including patient demographics, presence of NF1 or NF2, presenting symptoms, treatment strategy, latency from initial diagnosis of VS to the diagnosis of MPNST, histopathology, immunochemistry, recurrence, and survival were extracted and presented in tabular form. For cases that developed MPNST after resection of benign VS, the time of diagnosis of malignancy was the starting point for survival. Data were summarized with means, medians, and ranges, or frequency counts and percentages.

## Results

In the published literature since 1990, a total of 11 cases of MPNSTs arising from benign VS in the absence of irradiation or syndromic conditions have been reported ( Table 1 ). Patient ages ranged from 5 to 75 years, while the average age at diagnosis was 49. There was a minor female predominance ( *n* = 7). Four cases involved the left vestibulocochlear nerve, while seven were lateralized on the right. Common presenting symptoms included headache, ipsilateral cranial nerve deficits, and cerebellar symptoms such as gait disturbances. The most common presenting symptom was hearing loss on the ipsilateral side, which was present in seven cases. No cases identified a history of, and/or clinical examination features consistent with, neurofibromatosis type 1 or 2.

**Table 1:** Patient demographics and tumor characteristics

No.	Author, year, and citation no.	Age/Sex	Location	Symptoms	Initial pathology	Secondary pathology	Latency	Surgery	Chemo	Radiation	Survival
1	McLean et al, 1990 31	75 M	Right CN VIII	Hemianesthesia of body and face	VS	MPNST	11 months	STR	No	No	2 months
2	Han et al, 1992 19	47 F	Right CN VIII	Headache, paresthesia, hemiparesis	VS	Malignant triton tumor/MPNST	12 months	GTR	No	No	12 months
3	Son et al, 2001 32	33 F	Left CN VIII	HA, vertigo, gait disturbances, nystagmus, diplopia, hearing loss	VS	MPNST	2 months	GTR	No	Refused	Stable 1 year post-op
4	Gonzalez et al, 2007 33	43 F	Left CN VIII	Nausea, vomiting, gait disturbance, hearing loss	Malignant schwannoma	NA	7 months	GTR	No	Yes	8 months
5	Scheithauer et al, 2009 14	67 M	Right CN VIII	Hearing loss, neurologic decline	VS	MPNST	9 months	GTR	No	No	1 month
6		56 M	Right CN VIII	NA	VS	MPNST	7 months	STR	No	No	2 months
7		5 M	Left CNVIII	L facial palsy, hearingloss	VS and MPNST in same block	NA	NA	NOS	No	No	Alive on follow-up
8	Wei et al, 2012 34	41 F	Right CN VIII	Hypesthesia of left upper extremity, right hearing loss, R CNVII palsy (HB3)	VS and MPNST in same block	NA	NA	NOS	NA	NA	NA
9	Bashir et al, 2016 15	47 F	Right CN VIII	Tinnitus, unsteady gate, R facial numbness, decreased sensation on right face, R hearing loss	VS	MPNST	42 months	GTR	No	SRT (1.8 Gy per fraction, 30 fractions total)	Without any signs of tumor recurrence 9 months after irradiation
10	Belyaev et al, 2018 35	29 F	Right CN VIII	R hearing loss, HA, gait disturbance, decreased sensation R face, R CN7 palsy HB2, R cerebellar signs	VS	MPNST	6 months	STR	No	Yes	11 months
11	Present case	75 F	Left CN VIII	L facial numbness, loss of taste on L tongue, L sided CN VI and CN VII palsy, dysarthria, gait instability	VS and MPNST in same block	NA	NA	STR	No	No	1 month

Abbreviations: F, female; GTR, gross total resection; HA, headache; L, left; M, male; mo, months; NA, not applicable; NOS, not otherwise specified; R, right; STR, subtotal resection.

Gross total resection was accomplished in five cases, and subtotal resection was accomplished in four cases, while the resection status of the remaining two cases was not specified. Chemotherapy was not initiated in any of the patients. However, adjuvant radiation was initiated in two patients postoperatively. Follow-up data was limited, but seven patients expired within 1 year of resection.

Of the 11 cases, 7 had an established histological diagnosis of VS before resection of recurrent MPNST at the same site. In these cases, the latency from the initial diagnosis of VS to the diagnosis of MPNST ranged from 2 to 42 months. The average time of malignant transformation in these cases was 9.7 months. One case had a latency of 42 months, while all others were 9 months or less. Three cases, including the present case, had a histologically confirmed diagnosis of both VS and MPNST within the same block on initial resection.

Histological and immunochemical data were reviewed ( Table 2 ). There was a lack of uniformity among the reported characteristics, but all cases demonstrated increased cellularity and cytologic atypia. Necrosis was variable, and mitotic figures were present in all cases. When numerically reported, it ranged from 5 to 25 mitosis/high powered field (HPF). Out of 11 cases 8 demonstrated spindle-shaped cells, while round cell components and epithelioid cells were also present in the remaining cases. All cases reported S-100 immunochemical staining. S-100 was weak, scattered, or focal positive in 6 of 11 cases. One case was S-100 negative, and one was S-100 positive.

**Table 2:** Histopathology and immunochemistry

No.	Author, year, and citation no.	Age/Sex	Cellularity	Cytologic atypia	Necrosis	Mitosis/HPF	Histology	Immunotype
1	McLean et al, 1990 31	75 M	Increased	Pleomorphic spindle cells, hyperchromatic nuclei	Several	Many	Interlacing fasciculi of moderately pleomorphic spindle cells with hyperchromatic nuclei and frequent mitotic figures	S-100: scattered areas of positivity; vimentin: diffuse; desmin, EMA: negative; GFAP: negative
2	Han et al, 1992 19	47 F	High	Hyperchromatic nuclei, eosinophilic cytoplasm	NA	NA	Spindle cells and pleomorphic cells	S-100: positive; myoglobin: positive
3	Son et al, 2001 32	33 F	Increased	Hyperchromatic nuclei	NA	Frequent	Fascicular arrangement of atypical spindle cells	S-100: weakly and focally positive; p53 positive
4	Gonzalez et al, 2007 33	43 F	High	Pleomorphic nuclei, prominent nucleoli	Multiple foci	6–11	Atypical and markedly hypercellular spindle cells and bizarre epithelioid cells	S-100: focal; p53: 25–50%; neurofilament: negative; EMA: negative; GFAP: negative
5	Scheithauer et al, 2009 14	67 M	High	Severe	Absent	5	Round cell component	S-100: focal;collagen IV:diffuse; p53: high
6		56 M	Moderate	Severe	Focal	25	Spindle and small round cell component	S-100: neg;collagen IV:neg; p53:inconclusive
7		5 M	Moderate	Moderate	Absent	11	Epithelioid cells—parent tumor showed distinct Verocay body formation The MPNSTfeatured lobules and mucin deposition	S-100: positive; CAM 5.2; and EMA: neg
8	Wei et al, 2012 34	64 M	High	Hyperchromatic nuclei, scant cytoplasm	NA	Many	Two well-demarcated components: spindle-shaped cells forming intersecting fascicles with at least one Verocay body and second component with malignant features	First component: S-100 positive; second component MIB-1 up to 90%; both vimentin positive
9	Bashir et al, 2016 15	47 F	High	Focally pleomorphic cells	NA	15–20	Fascicular arrangement	S-100: positive focally; p53: strong/diffuse; MIB-1 up to 50%
10	Belyaev et al, 2018 35	29 F	High	Moderate pleomorphic cells, hyperchromatic nuclei	Several areas	Frequent	Interlacing fasciculi of spindle cells	S-100: scattered positivity; actin: positive in vessels walls; EMA: negative; desmin: negative
11	Present case	75 F	High	Severe	Focal	21	Spindle cell	S-100: diffuse positive and scattered positivity within the same block; pancytokeratin, GPAP and neurofilament negative

Abbreviations: EMA; F, female; GFAP; GPAP; HPF, high powered field; M, male; MPNST, malignant peripheral nerve sheath tumor.

## Discussion

Prior radiation is a known risk factor for MPNSTs. 12 14 Given the increasing number of patients receiving stereotactic radiosurgery for benign intracranial lesions, much of the literature over the past two decades has focused on radiation-induced malignancies. 16 While radiation-associated malignant transformation of VSs to MPNSTs has been well documented, few reports are available regarding MPNSTs secondary to spontaneous malignant transformation of VS. 16 17 18 Even rarer are reports of MPNSTs with rhabdomyoblastic differentiation (malignant triton tumor) arising from VS without prior radiation. 19

Establishing the diagnosis of MPNST arising from VS can be difficult as specific subtypes of VS, although still benign, may have similar histologic appearances to MPNSTs. The histopathology of high-grade MPNST lesions demonstrates geographically variable cellularity with long, straight fascicles that often intersect to form a herringbone pattern. The nuclei are elongated with irregular contours and indistinct cytoplasm. Necrosis is present and often widespread. Mitotic figures are often increased, greater than 10 per 10 high-power fields. The tumor edges are invasive and infiltrate into adjacent soft tissue structures. 20 In contrast, the histopathology of schwannomas consists of hyalinized blood vessels, collagenous encapsulation, and regionally variable cellularity. Other features that help to distinguish schwannomas include hemosiderin-laden macrophages, “ancient change,” and cystic degeneration. Subtypes include conventional, cellular, plexiform, epithelioid and “neuroblastoma-like,” and myxoid. Cellular schwannomas, a relatively rare schwannoma variant, can be easily misinterpreted as MPNSTs since they also show high cellularity, fascicular growth pattern, increased mitotic activity, and sometimes local destruction. Although these characteristics may prompt consideration of malignancy, cellular schwannoma is considered a benign neoplasm. Immunochemistry, specifically S-100 protein staining, is critical in differentiating between such benign neoplasms and MPNSTs. S-100 reactivity should be weak and/or focal in MPNSTs and strong and diffuse in cellular schwannomas. 21 However, the absence of S-100 protein reactivity is also common in MPNSTs, and therefore, S-100 negativity does not rule out the diagnosis. 22 Of note, Case 4 in our series demonstrated positive S-100 reactivity in a pathological block that had both VS and MPNST histology reported. Although the S-100 pattern of reactivity was not specified, the immunochemistry profile must be taken with caution and could represent a more benign pathology if, in fact, the S-100 staining was strong/diffuse rather than weak/focal.

In our case, the diagnosis of MPNST arising from VS transformation was established through a clinical history of a slow-growing mass and confirmed with histopathology and immunochemistry. Histopathology consisted of uniform spindle cells with hyperchromatic nuclei, high cellularity, and focal necrosis. Immunochemistry included S-100 diffuse positivity with scattered positivity within the same block. We further utilized loss of H3K27me3 expression to confirm the diagnosis of MPNST. 23 An additional panel of common immunochemistry reactivity was assessed to rule out other differentials. 24

Next-generation sequencing showed EGFR amplification, EGFRvIII exon 2–7 deletions, NF2 exon 12 splice donor mutation, and TP53 mutations in our patient sample. EGFR amplification has been reported in 24 to 28% of MPNSTs and is associated with poor prognosis. 25 26 The EGFRvIII variant, although more commonly associated with glioblastoma, has been rarely reported in MPNSTs and has been shown to contribute to increased tumor aggressiveness. 27 The NF2 exon 12 splice donor mutation is an interesting finding, as NF2 alterations are not typically associated with MPNSTs but rather with schwannomas and meningiomas. In a previous study, TP53 mutations were detected in 24% of MPNSTs and may contribute to tumor progression and treatment resistance. 25

These genetic alterations collectively suggest a complex molecular landscape for this MPNST. The EGFR amplification and EGFRvIII deletion indicate potential activation of the EGFR signaling pathway, which could drive tumor growth and invasion. 27 The TP53 mutations likely compromise p53's tumor suppressor function, potentially leading to genomic instability and resistance to apoptosis. 25 Although the significance of the NF2 mutation in this context is less clear, it may contribute to altered cell signaling and tumor development. 28 These findings highlight potential therapeutic targets, particularly EGFR, and underscore the importance of comprehensive molecular profiling in guiding personalized treatment strategies for MPNST patients.

Other studies have demonstrated that intracranial MPNSTs have unique risk factors and pathogenesis compared with MPNSTs of other locations. Approximately half of all MPNSTs of the trunk and extremities are associated with NF1. In these locations, MPNSTs frequently arise from a preexisting plexiform neurofibroma, while intracranial lesions are more likely to develop from neural tissue or from a precursor schwannoma. 29 Although previous reviews have shown that intracranial MPNSTs are more likely to develop in patients with neurofibromatosis (NF), our study found no such association. 30 Although this result is likely explained by our small case series, it may also be reflective of radiation as a precipitating risk factor in patients with NF. Further studies are needed to establish this relationship.

## Conclusion

Due to the limited number of reported cases of MPNST arising from VS without prior irradiation, there are still questions regarding its pathogenesis and pathological diagnosis. Our case demonstrates the malignant behavior of these tumors and the need for complete resection to optimize outcomes. We provide valuable additions to the literature to provide evidence for utilizing S-100 in diagnosing MPNST arising from schwannoma. Due to its rarity, more literature is needed to better understand this entity.
